# Bioengineered models to study tumor dormancy

**DOI:** 10.1186/s13036-018-0137-0

**Published:** 2019-01-10

**Authors:** Shreyas S. Rao, Raghu Vamsi Kondapaneni, Akshay A. Narkhede

**Affiliations:** Department of Chemical and Biological Engineering, The University of Alabama, Tuscaloosa, AL 35487-0203 USA

**Keywords:** Tumor dormancy, Tumor microenvironment, Metastasis, Bioengineered models

## Abstract

The onset of cancer metastasis is the defining event in cancer progression when the disease is considered lethal. The ability of metastatic cancer cells to stay dormant for extended time periods and reawaken at later stages leading to disease recurrence makes treatment of metastatic disease extremely challenging. The tumor microenvironment plays a critical role in deciding the ultimate fate of tumor cells, yet the mechanisms by which this occurs, including dormancy, is not well understood. This mini-review discusses bioengineered models inspired from tissue engineering strategies that mimic key aspects of the tumor microenvironment to study tumor dormancy. These models include biomaterial based three dimensional models, microfluidic based models, as well as bioreactor based models that incorporate relevant microenvironmental components such as extracellular matrix molecules, niche cells, or their combination to study microenvironmental regulation of tumor dormancy. Such biomimetic models provide suitable platforms to investigate the dormant niche, including cues that drive the dormant to proliferative transition in cancer cells. In addition, the potential of such model systems to advance research in the field of tumor dormancy is discussed.

## Introduction

The progression of cancer from the primary to the metastatic setting usually marks the transition to an incurable diagnosis [1]. Accumulating evidence suggests that disseminated tumor cells can stay in a dormant state for extended periods of time and could reawaken at a later stage resulting in disease relapse and often mortality [2]. For instance, greater than 67% of deaths from breast cancer occur beyond the 5-year survival window and disease recurrence is noted after almost a decade of being “cancer-free” in many patients [3, 4]. In addition, dormant tumor cells can also persist at the primary tumor site, following surgical resection of the primary tumor [5]. Tumor cells can also metastasize and stay dormant even prior to the evolution of the primary tumor [6]. While drug treatments exist, resistance to treatment is noted in many patients and the dormant/resistant tumor cells surviving treatment reactivate and contribute to disease progression at the primary and/or metastatic site [7] (i.e., in organs such as bone, liver, lung, and the brain). These observations highlight the need to understand the cellular and molecular mechanisms associated with tumor cell dormancy.

It is now well appreciated that the tumor microenvironment plays a significant role in controlling the dormant phenotype in tumor cells in addition to genetic alterations [2, 8–10]. In the context of metastatic disease, this is consistent with Paget’s “seed and soil” hypothesis proposed over a century ago, which states that metastasis occurs only when the organ environment (soil) is conducive to metastatic tumor cell (seed) growth [11–14]. Thus, experimental models to study and understand the mechanisms associated with dormancy must capture the bidirectional tumor cell-microenvironment interactions. In early work elucidating the role of microenvironment on tumor dormancy, Aguirre-Ghiso and colleagues showed that growth signals from fibronectin (an extracellular matrix (ECM) protein) via the urokinase plasminogen activator receptor (uPAR)-α_5_β_1_-integrin complex was critical, and thus reduction in the level of uPAR in human epidermoid cancer cells induced tumor dormancy when tested using standard tissue culture polystyrene (TCPS) substrates (routinely employed two dimensional (2D) culture models) in vitro as well as using mouse models in vivo [15]. Studies utilizing these models have also defined several key molecular features of tumor cell dormancy, including a high signaling ratio of p38/ERK [16–19].

A variety of in vivo mouse models, including genetically engineered mouse models, orthotropic /subcutaneous tumor models, tumor resection models, as well as experimental metastasis mouse models have been used to gain insight into tumor dormancy [20–23]. For instance, experimental metastasis mouse models have revealed the existence of a dormant state in cancer cells delivered to a metastatic organ site in vivo [24, 25]. However, mouse models provide limited control of the organ environment for controlled investigations. In addition, animal-animal variations, difficulties associated with imaging dormant cells in internal tissues, as well as high costs, can make the use of such models a challenging undertaking. In recent years, there has been a growing interest in utilizing components typically employed in tissue engineering (e.g., biomaterial scaffolds, tissue specific cells, and bioreactors) to study the tumor microenvironment and its role in governing tumor dormancy. These systems not only enable better recapitulation of the tumor microenvironment by capturing the relevant microenvironmental cues such as biophysical cues compared to the traditionally studied 2D culture models but also the study of tumor cell phenotype in a physiological relevant and controlled setting.

This review focuses on various tissue engineering inspired strategies that have been employed to elucidate microenvironmental regulation of tumor cell dormancy. In particular, we discuss biomaterial based models, microfluidic based models, as well as bioreactor based models and how these bioengineered models have been utilized to study the dormant phenotype as well as the transition from a dormant to proliferative phenotype in cancer cells. Collectively, such microenvironment mimicking model systems provide useful tools to probe the dormant niche as well as elucidate the molecular mechanisms regulating tumor dormancy.

## Bioengineered models mimicking the tumor microenvironment to study tumor cell dormancy

### Biomaterial based models

Biomaterial scaffolds commonly employed in tissue engineering such as hydrogels, porous scaffolds, and electrospun fibrous scaffolds have been used as models to study tumor cell dormancy. Such three dimensional (3D) culture systems could be engineered to mimic specific features of the tumor microenvironment (e.g., stiffness, topography) as well as incorporate other relevant noncancerous cells. In this section, we discuss the various types of biomaterial based models that have been employed to study microenvironmental regulation of tumor dormancy.

#### Natural biomaterial based models

A variety of natural biomaterials have been used to study tumor cell dormancy and maintenance of this state via targeting the cytoskeletal organization [26], incorporating relevant niche cells [27, 28], modulation of stiffness [29], or via modulation of signaling pathways (e.g., Src family kinase (SFK) inhibition [30]). Specifically, hydrogels composed of Collagen-I [31], hyaluronic acid [32], fibrin [29], and Matrigel [26, 30, 31, 33] have been employed (studies summarized in Table 1). Barkan et al., utilized Basement Membrane Matrix (BME) (or Matrigel) and found that this matrix maintained the dormant state of D2.0R cancer cells that were observed to be dormant in vivo as opposed to traditionally studied 2D models (e.g., TCPS) and that the transition to the proliferative state was mediated via β-1 integrin signaling [26]. Further, myosin light chain kinase (MLCK) activation was also necessary for this transition as inhibition of MLCK or β-1 integrin impeded the dormant to proliferative state transition. Similarly, A549 lung cancer cells cultured in Matrigel underwent dormancy and exhibited drug resistance compared to standard 2D culture (TCPS) [34].

**Table 1:** Summary of the studies utilizing bioengineered models to study tumor dormancy

Type of Cancer	Cancer Cell Lines	Niche cells	Biomaterial	Key Findings	References
Biomaterial based models					
Breast Cancer and Osteosarcoma	D2.0R, D2A1, MCF-7, MDA-MB-231, 4 T1, K7 M2, K7M2AS1.46	–	Basement Membrane Matrix	Inhibition of MLCK maintained the dormant phenotype of cancer cells and reduced metastatic outgrowth	Barkan et al., [26]
Breast Cancer and Osteosarcoma	D2.0R, D2A1, MCF-7, MDA-MB-231, K7 M2, K7M2AS1.46	–	Basement Membrane Matrix	Cells can be maintained in dormant state by inhibiting MLCK phosphorylation	Barkan et al., [33]
Breast Cancer	D2.0R, D2A1	–	Basement Membrane Matrix	Collagen-I enriched fibrotic environment revoked dormant phenotype in cancer cells	Barkan et al., [35]
Breast Cancer and Osteosarcoma	MDA-MB-231, K7 M2, 4 T1, D2.0R, D2A1	–	Basement Membrane Matrix	Joint inhibition of SFK and MEK1/2 prevented switch from dormant to proliferative phenotype in cancer cells while simultaneously suppressing their survival	EL Touny et al., [30]
Breast Cancer	MDA-MB-231	HMEC-1	Hyaluronic acid hydrogel	Hyaluronic acid environment lowers the ERK/p38 ratio in cancer cells in 3D co-culture, which is indicative of a dormant phenotype	Kassim et al., [32]
Breast, Prostate and Ovarian Cancer	MCF-7, MDA-MB-231, MDA-MB-468, OVCAR-5, MCF10ADCIS.COM, LnCAP	–	Silica-PEG hydrogel	Physical immobilization of cancer cells in a stiff mechanical environment induces dormancy	Preciado et al., [54]
Melanoma, Liver, Lung and Breast Cancer	B16,4 T1, H22, A375, A549, HepG2	–	Fibrin gel	Nuclear translocation of Cdc42 in a stiff mechanical environment induces dormancy in B16 cells by activating Cdc42-tet2 pathway	Liu et al., [29]
Breast Cancer	MDA-MB-231, MCF-7, T4–2	HUVEC, Lung Fibroblasts, Bone marrow mesenchymal stem cells	Laminin rich ECM	Stable microvascular endothelium induces quiescent phenotype in disseminated tumor cells (DTC) which is mediated by Thrombospondin-1 whereas, sprouting endothelium promote proliferation in DTCs by producing periostin & TGF-β1	Ghajar et al., [28]
Breast Cancer	SUM159, SUM149, MDA-MB-231, MDA-MB-435, BT474, MCF-7, T47D, ZR75–1	HUVEC, Bone marrow derived mesenchymal cells (HS-5), hFOB, BMSC	Collagen biomatrix	Niche cells HUVEC, HS-5, and hFOB induce quiescence in cancer cells and this phenotype can be reversed by inhibiting p38, ALK5, and RTK	Marlow et al., [27]
Breast and Ovarian Cancer	MCF-7, MDA-MB-231, OVCAR-3	–	Collagen gel	Chemically induced hypoxia through CoCl_2_ treatment induces dormancy in MCF-7 cells by increasing p38/pERK ratio	Lee et al., [51]
Breast, Colon and Pancreatic Cancer	MDA-MB-231, HCT-116,CFPAC-1	–	Collagen gel	Stiff mechanical environment coaxed the cancer cells towards dormancy which resulted in increased drug resistance	Fang et al., [31]
Liver Cancer	Huh7, HepG2	–	PA gel	Soft environments induced dormancy in cancer cells by downregulating the phosphorylation of FAK, ERK and STAT3 resulting in increased drug resistance and stem-like characteristics	Schrader et al., [53]
Prostate Cancer	M12, P69, M12mac25, LNCaP C4–2	–	pHEMA scaffolds	pHEMA scaffolds suppressed the proliferative phenotype in LNCaP cells in vivo whereas, they promoted the growth of M12mac25 cells by triggering the dormant to proliferative switch	Long et al., [55]
Breast Cancer	MDA-MB-231, T47D	–	PCL fibrous scaffold	Cancer cells cultured on PCL scaffolds and treated with carboplatin exhibited a quiescent phenotype	Guiro et al., [56]
Bladder, Prostate, Lung, Breast Cancer and Neuroblastoma	T24, UMUC-3, PC3, PC3-PSMA, MCF-7, MDA-MB-231, H1975, SH-SY5Y	NIH3T3 mouse fibroblasts, BJ-5ta human foreskin fibroblasts & WPMY-1 human prostate stromal cells	Amikagel	Bladder cancer cells on ~ 215 kPa Amikagels were growth arrested and resistant to docetaxel	Pavan Grandhi et al., [57]
Lung Cancer	A549	–	Matrigel	Matrigel induced dormancy in A549 cells via inhibition of PI3K/Akt and ERK1/2 pathways.	Keeratichmroen et al., [34]
Bladder Cancer	SV-HUC-1, TCCSUP, RT4, J82, 253JP, 253JB-V	–	Matrigel, SIS gel	SIS gel suppressed the growth and malignant phenotype of bladder cancer cells by driving them towards dormancy whereas, Matrigel supported the malignant and invasive phenotype	Hurst et al., [46]
Bladder Cancer	SV-HUC-1, TCCSUP, RT4, J82, 253JP, 253JB-V	–	Matrigel, SIS gel	SIS gel suppresses malignant phenotype in cancer cells as indicated by gene expression pattern whereas Matrigel promotes the expression of growth associated genes	Dozmorov et al., [47]
Bladder Cancer	J82, JB-V	–	Matrigel, Collagen, SIS gel	Dormant phenotype was observed in J82 or JB-V cells injected with SIS gel in nude mice	Hurst et al., [48]
Breast, Bladder, Prostate, Pancreatic, Gastric Cancer and Glioblastoma	MDA-MB-231, MDA-MB-435, MDA-MB-235, 4 T1, J82, PC-3, DU145, U251, AGS, Capan-1	–	SIS gel	Identified drugs to target dormant cells	Hurst et al., [49]
Microfluidic based models					
Breast Cancer	MDA-MB-231	Human hepatocytes, NPCs	PEG hydrogel	Dormant phenotype was observed in cancer cells co-cultured with hepatic niche on the liver-chip incorporating PEG hydrogel	Clark et al., [61]
Breast Cancer	MDA-MB-231, MCF-7	Human hepatocytes, NPCs	–	A subpopulation of cancer cells underwent dormancy on the liver chip and this was associated with a differential cytokine profile	Wheeler et al., [58]
Breast Cancer	MDA-MB-231, MCF-7	Human mammary epithelial cells, human hepatic stellate cells (LX-1, LX-2, TWNT-1), rat hepatic stellate cells (HSC-T6) human endothelial cells (TMNK-1)	–	IL-8 revoked the hepatocyte induced dormancy in MDA-MB-231 cells	Khazali et al., [59]
Breast Cancer	MDA-MB-231	Human hepatocytes, NPCs	–	Introduction of inflammatory stimuli promoted growth of dormant cancer cells	Clark et al., [60]
Lung Cancer	H1975	Human primary airway and alveolar epithelial cells, Human lung microvascular epithelial cells	–	Breathing motion incorporated in engineered lung chip decreased cancer cell growth leading to a dormant phenotype	Hassel et al., [62]
Bioreactor based model					
Breast Cancer	MDA-MB-231, MCF-7, MDA-MB-231BRMS1	Mouse MC3T3-E1 osteoblasts, Normal Human Osteoblast cells	–	Remodeling cytokines TNFα and IL-1β promoted metastatic outgrowth of dormant MDA-MB-231BRMS1 cells	Sosnoski et al., [63]

*MLCK* Myosin light chain kinase, *SFK* Src family kinases, *MEK* Mitogen activated protein kinases, *ERK* Extracellular-signal regulated kinases, *Cdc42* Cell division control protein 42, *Tet-2* Tet methylcytosine dioxygenase 2, *TGF-β1* Transforming growth factor beta 1, *ECM* Extracellular matrix, *HUVEC* Human umbilical vein endothelial cells, *FAK* Focal adhesion kinase, *STAT3* Signal transducer and activator of transcription 3, *BMSC* Bone marrow stromal cells, *RTK* Receptor tyrosine kinase, *pHEMA* poly (2-hydroxyethyl methacrylate), *PCL* Polycaprolactone, *PEG* Polyethylene glycol, *PA* Polyacrylamide, *SIS* Small intestine submucosa, *HMEC* Human microvascular endothelial cells, *TNFα* Tumor necrosis factor alpha, *NPCs* Nonparenchymal cells, *hFOB* Human fetal osteoblasts, *IL* Interleukin, *PI3K* Phosphoinositide 3-kinase, *Akt* Protein kinase B

In contrast to BME inducing a dormant state, incorporating Collagen-I within BME lead to a proliferative phenotype in dormant mouse breast cancer D2.0R cells in vitro [35]. Activation of β-1 integrin was responsible for the emergence of this phenotype and thus inhibiting β-1 integrin and the associated downstream signaling pathway components (Src, extracellular-signal regulated kinase (ERK), or MLCK) significantly inhibited proliferation. Modulation of signaling pathways to control the dormant vs. proliferative phenotype has also been investigated using natural biomaterial based models. Specifically, SFK inhibition caused localization of p27 (cyclin dependent kinase inhibitor) to the nucleus and inhibited proliferation that was induced by incorporating Collagen-I into BME [30]. Further, combined targeting of SFK and mitogen activated protein kinase (MEK) was shown to induce apoptosis in dormant cancer cells, thereby demonstrating the efficacy and potential of this combinatorial treatment for treating recurrent disease.

Niche cells present in the tumor microenvironment have been incorporated into natural biomaterial scaffolds to create a model of dormancy for bone metastatic breast cancer cells. For example, Marlow et al., employed a 3D collagen biomatrix that were seeded with either primary bone marrow stromal cells (BMSC) or a mix of osteoblasts, mesenchymal, and endothelial cell lines (BMCL-Bone marrow cell lines) [27]. In this system, breast cancer cells co-cultured with BMSCs proliferated whereas those cultured with BMCL remained in a dormant state and this phenomenon was observed both in vitro and in vivo. Moreover, breast cancer cells retrieved from BMCL co-cultures began proliferating when co-cultured with BMSCs. The dormant state observed in this model was also reversible when p38, and receptor tyrosine kinase (RTK) (pathways involved in dormancy [36–38]) was inhibited. These observations were also validated in vivo by subcutaneously implanting cell-laden biomaterial constructs in murine models. Such “hybrid *in vivo* models” wherein biomaterial scaffolds are integrated with murine models have been recently utilized in several investigations to study the metastatic niche [39–45]. Similarly, Ghajar et al., demonstrated that endothelial cells influenced the dormant phenotype in breast cancer cells in a laminin-rich ECM [28]. Specifically, established or stable endothelium induced a dormant state via endothelial-derived thrombospondin-1 (TSP-1). In contrast, the authors showed that cancer cell growth was accelerated at sprouting neovascular tips (i.e., sprouting endothelium), which was associated with enhanced expression of Transforming growth factor beta 1 (TGF-β1) and periostin, and with the loss of TSP-1. In a hyaluronic acid hydrogel model, when breast cancer cells were co-cultured with a human microvascular endothelial cell line (HMEC-1), expression of ERK/p38 was reduced in co-culture compared to breast cancer cell monoculture indicating the emergence of a dormant state in breast cancer cells [32].

Similar to the utilization of Matrigel, Hurst et al., [46] utilized SIS gel (derived from small intestine submucosa (SIS) representative of a normal basement membrane matrix) to study phenotype regulation in bladder cancer cells and compared it with Matrigel (representative of a remodeled tumor matrix). In these studies, Matrigel promoted a more invasive phenotype as opposed to a non-aggressive phenotype that was observed in the SIS gel. Further, cells isolated from Matrigel when grown on SIS gel demonstrated growth characteristics similar to cells grown on SIS gel and vice versa demonstrating that this phenotype regulation was dependent on the gel composition. These results were further supported via comparative gene expression studies [47]. In a follow up study, these observations were further validated using hybrid in vivo models [48]. In particular, when J82 or JB-V bladder cancer cells were subcutaneously injected with SIS gel in nude mice, cancer cells were observed to be in a dormant state with no sign of tumor formation. However, in some cases, cells transitioned from a dormant to a proliferative state. Tumor growth was noted in 40% of SIS gel xenografts following a 4–18 week dormancy period. Specifically, the transition from a dormant to a proliferative phenotype was dependent on the number of implanted tumor cells, with tumors more likely to form when more than 3 million tumor cells were implanted [48]. These models have also been utilized to identify therapeutics that target dormant cells [49] .

**Fig. 1:**
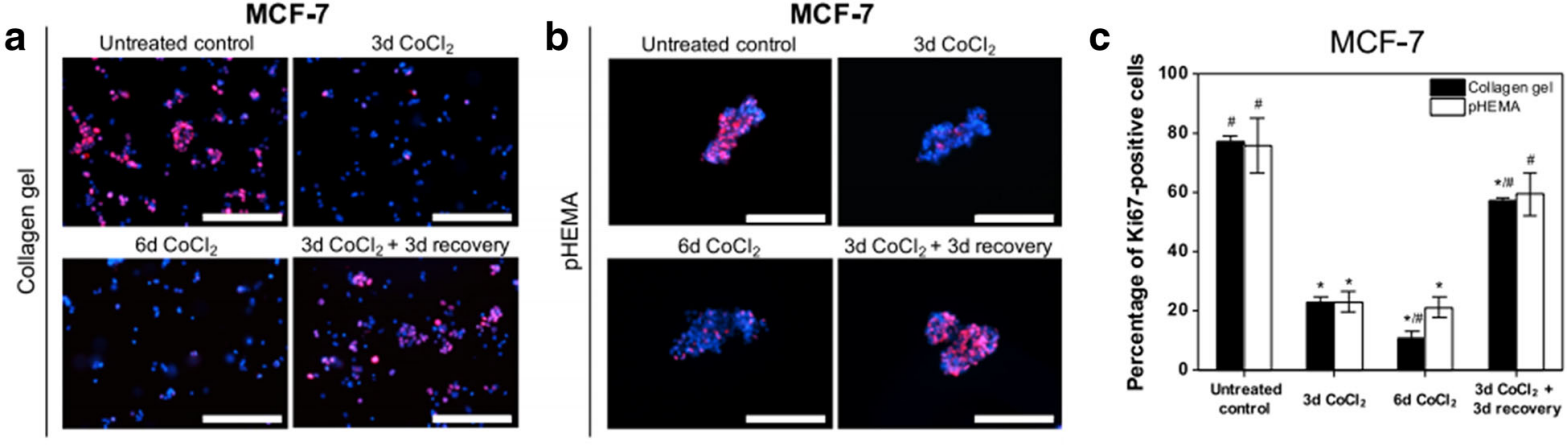
In a Collagen hydrogel incorporating hypoxia mimicking agent CoCl_2_ (300 μM) or pHEMA coated culture plates, MCF7 breast cancer cells exhibited a dormant phenotype, which was reversible after treatment with CoCl_2_ free growth media. Fluorescence images of MCF7 cells stained for Ki67 (red) and nuclei (blue) for untreated control, 3 day treatment with CoCl_2_, 6 day treatment with CoCl_2_ and 3 day treatment with CoCl_2_ followed by 3 day recovery period in (**a**) Collagen hydrogels and (**b**) pHEMA coated culture plates and (**c**) quantification of Ki-67 status in these conditions. Scale bar = 200 μm. Figure taken from [51] and reprinted with permission of BioMed Central (Springer Nature)

Hypoxia, a characteristic feature of the tumor microenvironment [50], has also been incorporated with natural biomaterials such as Collagen to develop dormancy models. For example, Lee et al., utilized cobalt chloride (CoCl_2_) (a hypoxia mimicking agent) with Collagen gels to induce dormancy in breast cancer cells [51]. They found that MCF-7 breast cancer cells exhibited a dormant phenotype in this model system and this phenotype was reversible when the cells were grown in CoCl_2_ free media. These results were also observed when the cells were grown on non-adhesive poly(2-hydroxyethyl methacrylate) (pHEMA) coated tissue culture plates (Fig. 1).

More recently, fibrin gels were employed to elucidate the impact of matrix stiffness on tumor cell dormancy. Specifically, Liu et al., employed [29] fibrin gels of 90, 450 and 1050 Pa bracketing the range of stiffness noted for many tissues (100–3000 Pa [52]). In this system, murine B16 & human melanoma A375 cells embedded within 1050 Pa fibrin gels remained dormant as opposed to those in 90 Pa gels. This induced dormancy was reversible, as cells isolated from 1050 Pa fibrin gel proliferated when cultured in 90 Pa gels. Maintenance of the dormant state with increasing stiffness in this system was mediated via translocation of cell division control protein 42 (Cdc42) from the cytosol to the nucleus, in turn, promoting tet methylcytosine dioxygenase 2 (Tet-2) expression, and subsequently activating cell-cycle inhibiting p21 and p27 genes.

#### Synthetic biomaterial based models

In addition to natural biomaterial-based models, synthetic biomaterial systems such as polyacrylamide (PA), silica-polyethylene glycol (silica-PEG), polycaprolactone (PCL), and pHEMA have been utilized to study the impact of tumor microenvironment on the dormant phenotype. Synthetic biomaterials provide a highly tunable platform and are more reproducible compared to natural biomaterial-based models. Schrader and colleagues utilized PA hydrogels to study the influence of matrix stiffness on the behavior of hepatocellular carcinoma cells [53]. They found these cancer cells cultured on stiff hydrogels (12 kPa) rapidly proliferated compared to soft hydrogels (1 kPa) as indicated via increased Ki67 (a proliferation marker) positivity, with the soft hydrogels promoting a more dormant-like phenotype. Inhibition of β1-integrin or Focal adhesion kinase (FAK) significantly reduced Ki-67 status on stiff hydrogels (12 kPa), thereby implicating these pathways in the observed cellular response.

Physical immobilization of cancer cells in synthetic biomaterials has also been shown to induce a dormant phenotype in cancer cells. For instance, MCF-7 breast cancer cells encapsulated in a porous silica-PEG hydrogel system underwent cell-cycle arrest, but resumed proliferation when they were retrieved from the hydrogel and cultured on TCPS [54]. Similarly, Long et al., employed sphere-templated porous pHEMA hydrogels to develop prostate cancer xenografts [55]. Using this system, they demonstrated that M12mac25 prostate cancer cells subcutaneously inoculated into athymic nude mice using Matrigel stayed largely dormant. However, with pHEMA scaffolds (with or without Matrigel) tumor formation was noted providing a model of dormancy escape in prostate cancer cells.

In addition to hydrogels, synthetic electrospun fiber-based biomaterials have been used to study tumor dormancy. To this end, random or aligned electrospun PCL fibrous scaffolds were used to examine the behavior of Carboplatin (a chemotherapy) treated vs. non treated breast cancer cells [56]. Non treated breast cancer cells exhibited a more dormant phenotype on fibrous scaffolds as evidenced using cell cycle analysis whereas the treated breast cancer cells exhibited this phenotype when cultured on fibrous scaffolds as well as TCPS.

#### Semi-synthetic biomaterial based models

Semi-synthetic scaffolds fabricated using a combination of natural and synthetic materials have also been investigated to develop models of tumor dormancy. For example, Pavan Grandhi et al., utilized amikacin hydrate and poly (ethylene glycol) diglycidyl ether (PEGDE) to develop a new hydrogel termed as “Amikagel” that was used to study dormancy in bladder cancer [57]. They found that 90% of T24 bladder cancer cells cultured on ~ 215 kPa Amikagels were cell cycle arrested in G0/G1 phase and were resistant to chemotherapeutic drugs such as docetaxel. However, when cells from the ~ 215 kPa Amikagels were transferred to ~ 36 kPa Amikagels, a sub-population of cells escaped dormancy and began proliferating. Overall, such biomimetic biomaterial based models provide useful tools to better understand the dormant niche. For instance, biomaterial based models are well suited to probe the impact of biophysical cues (such as matrix stiffness) on tumor dormancy versus traditional 2D culture models. These tools would also subsequently allow the study of molecular mechanisms governing the dormant phenotype as well as the dormant-to-proliferative switch.

### Microfluidic based models

**Fig. 2:**
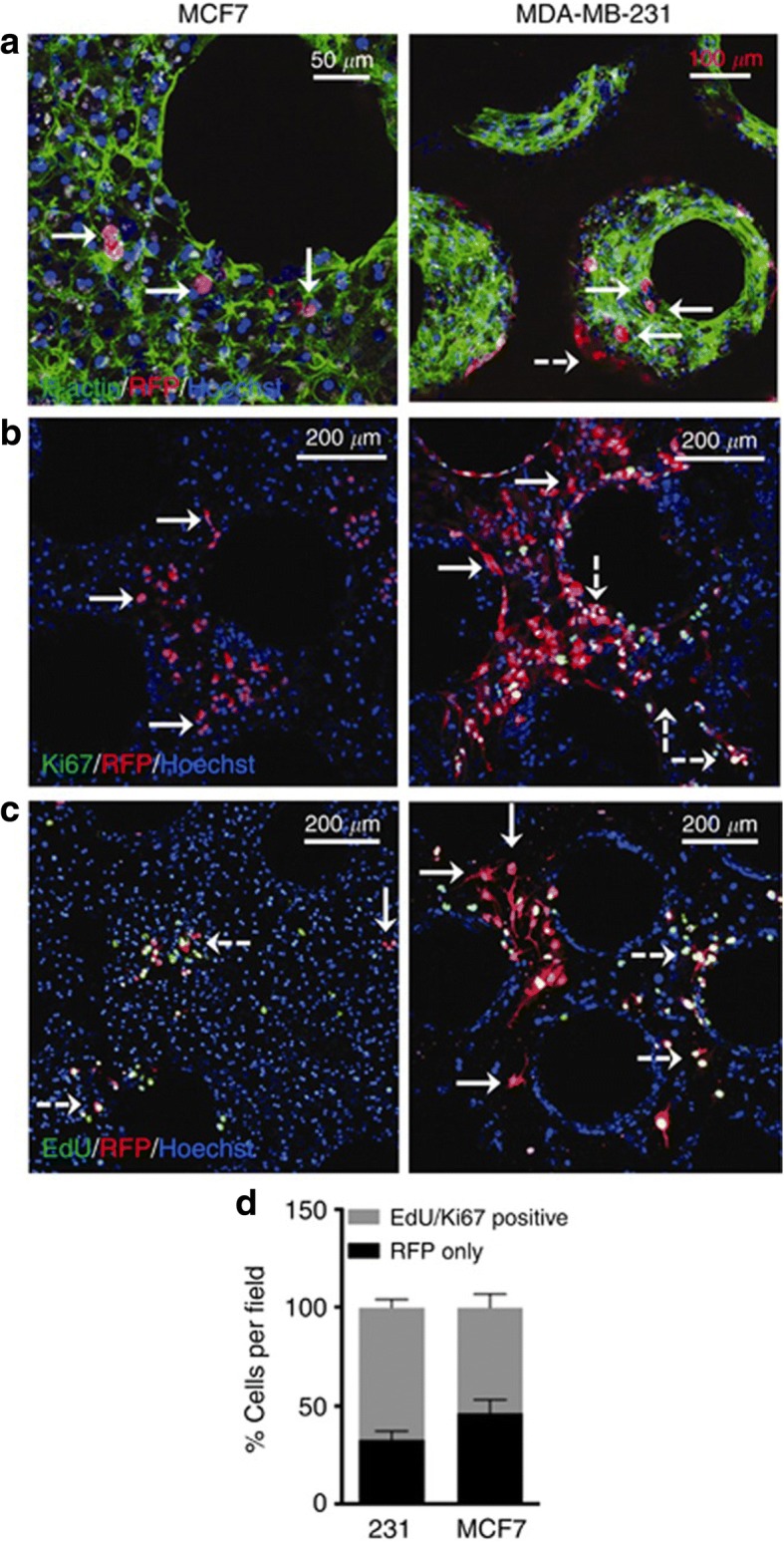
In a liver chip model, a subpopulation of MCF7 and MDA-MB-231 breast cancer cells underwent growth arrest. **a** Fluorescence image of MCF7 and MDA-MB-231 cells seeded with hepatocytes and non-parenchymal cells (F-Actin = green; Hoechst = blue, tumor cells = red (RFP) (**b**) Ki67 staining (green) and (**c**) EdU staining (green) of tumor cells and (**d**) Quantification of Ki67 and EdU status for both cell lines. Solid arrows indicate dormant cells and dashed white arrows indicate proliferating cells. Figure taken from [58] and reprinted with permission of Springer Nature

Microfluidic based models have also been used to study tumor dormancy. Such models allow for incorporation of nutrient/growth factor gradients. In addition, niche cells present in the tumor microenvironment are also typically incorporated in these models. One of the microfluidic based models is the commercially available LiverChip® wherein hepatocytes and non-parenchymal cells (NPCs) can be co-cultured to form an ex vivo microphysiologic model of the liver that could be used to study dormancy in cancer cells, including those that metastasize to the liver [58]. In this system, hepatocytes can be cultured for ~ 15 days without losing their functionality. This setup also contains an oxygen sensor and micro-reactor pumps to control the flow of nutrients and growth factors. In this system, a sub population of MDA-MB-231 and MCF7 breast cancer cells underwent dormancy (Fig. 2) that was associated with an increase in cancer attenuation signals (i.e., follistatin) and decrease in the pro-inflammatory signals (Insulin like growth factor binding protein 1 (IGFBP-1), Macrophage inflammatory protein 1 alpha (MIP-1α), Monocyte chemoattractant protein (MCP-1) & Interleukin-6 (IL-6)) for MDA-MB-231 cells, whereas in the case of MCF-7 cells, increase in cancer associated (e.g., Vascular endothelial growth factor A (VEGF-A), Epidermal growth factor (EGF)) and pro-inflammatory signals (IL-6, MCP-1) was noted. More recently, Khazali et al., tested if inflammatory signals present in the hepatic niche (from hepatic stellate cells) stimulated escape from the dormancy phenotype using the LiverChip® [59]. Indeed, introduction of IL-8 promoted proliferation of otherwise dormant MDA-MB-231 breast cancer cells as tested using EdU incorporation assay. This was also associated with an increase in phosphorylated ERK levels. Similarly, Clark et al., demonstrated that introduction of an inflammatory stimuli such as EGF or lipopolysaccharide (LPS) promoted proliferation of dormant MDA-MB-231 breast cancer cells [60].

Biomaterial scaffolds have also been incorporated into microfluidic based models for studies of tumor dormancy. For example, a PEG based hydrogel was incorporated into the liver microphysiological system by Clark et al., in a follow up study [61]. In this model, MDA-MB-231 breast cancer cells exhibited a dormant phenotype on the PEG based hydrogel as compared to the polystyrene. Further, these cells were also found to be resistant to high doses of chemotherapy drugs such as Cisplatin and Doxorubicin on the hydrogel as opposed to polystyrene supported cultures.

In addition to breast cancer, microfluidics based models have been employed to study dormancy versus growth in lung cancer. A lung cancer-on-a-chip, specifically, lung airway chip and lung alveolus chip, was developed by Hassell and colleagues utilizing microfluidics [62]. Both chips utilize a two channel microfluidic set-up separated via a porous membrane coated with ECM proteins and incorporate airway or lung alveolar epithelial cells interfaced with endothelial cells. In this model, they found that non-small-cell lung cancer cells stayed relatively dormant in the lung airway chip as opposed to the lung alveolus chip wherein significant growth was observed.

### Bioreactor based models

**Fig. 3:**
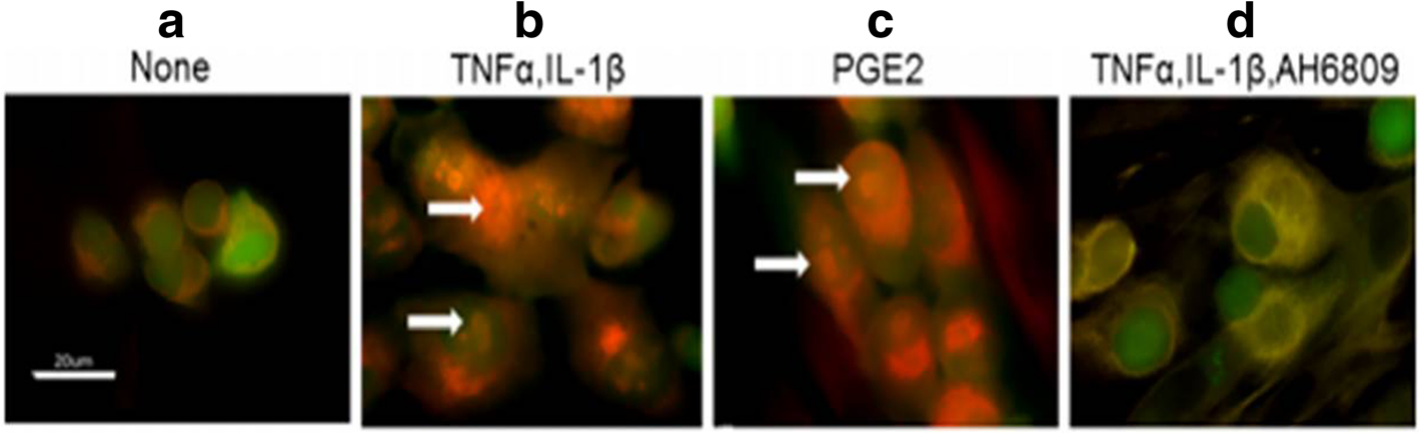
In a bioreactor model, addition of TNFα and IL-β1 or PGE2 enabled proliferation of MDA-MB-231BRMS1 cells that were otherwise growth arrested as indicated via Ki67 staining. Fluorescence images of cells stained for Ki67 in (**a**) untreated control, (**b**) TNFα and IL-β1 treatment, (**c**) PGE2 treatment, and (**d**) TNFα, IL-1β and AH6809 (PGE2 receptor inhibitor) treatment conditions. White arrows indicate positive nuclear Ki67 staining. Scale bar = 20 μm. Figure taken from [63] and reprinted with permission of Springer Nature

In addition to biomaterial and microfluidic based models, bioreactor based models have been used to investigate dormancy. Niche cells are also incorporated in such models as they allow long term culture. Such a model was utilized by Sosnoski et al. [63], to study breast cancer cell dormancy in a bone mimetic environment as breast cancer cells are known to metastasize to the bone [64, 65]. In this model, a bioreactor was employed to culture bone cells (murine MC3T3-E1 and human osteoblast cells) for up to 120 days. During this culture period, osteoblasts generated tissue that contained 6 or more layers of cells mimicking the pericellular environment [66]. Two month old bioreactor cultures were employed to which cytokines involved in bone remodeling were added, followed by addition of breast cancer cells. Specifically, a metastasis-suppressed MDA-MB-231BRMS1 human breast cancer cell line was used. Addition of cytokines tumor necrosis factor alpha (TNFα) and IL-1β to the bioreactor co-cultures allowed these cells to grow, which otherwise were largely growth arrested. This behavior was also seen when prostaglandin E2 (PGE2) was added to the cultures and addition of PGE2 receptor inhibitor suppressed tumor cell proliferation as seen via Ki67 staining (Fig. 3). The authors also observed a significant enhancement in focal adhesion kinase plaque formation in cancer cells in TNFα and IL-1β treated bioreactor co-cultures. While only few studies have utilized bioreactor based platforms, such platforms provide a better in vitro model system for co-culturing cancer cells as well as niche cells (e.g., breast cancer cells and osteoblasts) for longer time periods. This is advantageous as cancer cells typically stay dormant for extended periods of time in vivo and such models could be employed to capture these characteristic features.

## Conclusions and perspectives

To elucidate the mechanisms governing dormancy, bioengineered models such as biomaterials, microfluidics, and bioreactor- based models are being increasingly utilized as biomimetic in vitro culture systems to model tumor dormancy. Unlike in vivo models, bioengineered models highlighted herein allow us to pursue a reductionist approach and thereby study how individual microenvironmental cues regulate dormancy in cancer cells owing to their versatility and tunability. To this end, these models have been largely utilized to investigate the impact of mechanical cues, biochemical cues, as well as cellular cues on tumor cell dormancy. Specifically, the cellular cues incorporated in current models largely consist of stromal and vascular cells. However, in addition to stromal and vascular cells, immune cells play a key role in cancer progression and metastasis [67–69]. Future studies should aim at incorporating immune cells such as macrophages in bioengineered models for studying immune-mediated dormancy. Further, 3D in vitro models have recently been utilized to study the microenvironmental regulation of stem-like phenotype in cancer cells [70]. There are striking parallels between cancer stem-like cells (CSCs) and dormant cancer cells. For instance, CSCs exhibit behaviors similar to dormant cancer cells such as increased drug resistance and the ability to repopulate the tumor mass in response to certain microenvironmental cues [71]. However, it is not clear whether they belong to the same dormant population or consist of a distinct population. Bioengineered models could be employed to clarify the extent of overlap between the cancer stem-like phenotype and the dormant phenotype. In addition, these models could be utilized to study the role of fundamental biological processes such as epithelial-to-mesenchymal transition and mesenchymal-to-epithelial transition in regulating cancer cell dormancy as they are known to be involved in cancer metastasis [72, 73]. Finally, current bioengineered models largely focus on single cell (cellular) dormancy, however, balance between proliferation and apoptosis could also lead to tumor dormancy (also called tumor mass dormancy) [2, 74]. It would be worthwhile to model these mechanisms in vitro using biomimetic culture systems as it will further our understanding of tumor mass dormancy. Overall, in the short term, bioengineered models could provide key scientific insight into microenvironmental regulation of the dormant phenotype and, in the long term, may enable the development of therapeutic strategies targeting dormant or active metastatic disease.

## Abbreviations

Akt: Protein kinase B
BMCL: Bone marrow cell lines
BME: Basement membrane matrix
BMSC: Bone marrow stromal cells
Cdc42: Cell division control protein 42
CSCs: Cancer stem cells
ECM: Extracellular matrix
EGF: Epidermal growth factor
ERK: Extracellular-signal regulated kinase
FAK: Focal adhesion kinase
hFOB: Human fetal osteoblasts
HMEC: Human microvascular endothelial cells
HUVEC: Human umbilical vein endothelial cells
IGFBP-1: Insulin like growth factor binding protein 1
IL: Interleukin
ILK: Integrin linked kinase
LPS: Lipopolysaccharide
MCP-1: Monocyte chemoattractant protein 1
MEK: Mitogen-activated protein kinase
MIP-1α: Macrophage inflammatory protein 1 alpha
MLCK: Myosin light chain kinase
NPCs: Nonparenchymal cells
PA: Polyacrylamide
PCL: Polycaprolactone
PEG: Polyethylene glycol
PEGDE: Poly (ethylene glycol) diglycidyl ether
PGE2: Prostaglandin E2
pHEMA: poly (2-hydroxyethyl methacrylate)
PI3K: Phosphoinositide 3-kinase
RTK: Receptor tyrosine kinase
SFK: Src family kinases
SIS: Small intestine submucosa
STAT3: Signal transducer and activator of transcription 3
TCPS: Tissue culture polystyrene
Tet-2: tet methylcytosine dioxygenase 2
TGF-β1: Transforming growth factor beta 1
TNFα: Tumor necrosis factor alpha
TSP-1: Thrombospondin-1
uPAR: Urokinase plasminogen activator receptor
VEGF-A: Vascular endothelial growth factor A
